# Surgical approaches in hemiarthroplasty for hip fracture: results of 48,222 hemiarthroplasties in the Norwegian Hip Fracture Register

**DOI:** 10.1302/2633-1462.610.BJO-2025-0099.R1

**Published:** 2025-10-22

**Authors:** Raymond Tellefsen, Torbjørn B. Kristensen, Eva H. Dybvik, Jan-Erik Gjertsen, Lars Nordsletten, Terje Ugland, Håvard Visnes, Lene B. Solberg

**Affiliations:** 1 Division of Orthopaedic Surgery, Oslo University Hospital, Oslo, Norway; 2 Institute of Clinical Medicine, University of Oslo, Oslo, Norway; 3 The Norwegian Hip Fracture Register, Department of Orthopaedic Surgery, Haukeland University Hospital, Bergen, Norway; 4 Department of Clinical Medicine, University of Bergen, Bergen, Norway; 5 Division of Orthopaedic Surgery, Sorlandet Hospital Kristiansand, Kristiansand, Norway

**Keywords:** Femoral neck fracture, Hemiarthroplasty, Surgical approach, hemiarthroplasties, hip fractures, surgical approaches, revision surgery, visual analogue scale, EQ-5D-3L, intraoperative complications, infections, posterior approach, Cox regression model

## Abstract

**Aims:**

The aim of this study was to compare the direct lateral approach (DLA) with the anterolateral approach (ALA) and posterior approach (PA) using data on hemiarthroplasties (HAs) reported to the Norwegian Hip Fracture Register. The primary endpoint was reoperations within 12 months post-surgery. Secondary endpoints included mortality, patient-reported outcome measures (PROMs; EuroQol five-dimension three-level questionnaire (EQ-5D-3L) and EuroQol visual analogue scale (EQ-VAS)), and intraoperative complications.

**Methods:**

A total of 39,905 HA patients aged 60 years or older who were operated on using DLA from January 2005 to December 2023 were compared to 2,813 patients operated on with ALA and 5,504 with PA in the same period. Hazard rate ratios (HRRs) for reoperations and mortality were calculated using Cox regression adjusted for age, sex, American Society of Anesthesiologists classification, cognitive status, and fixation method. Patients reported EQ-5D-3L and EQ-VAS 12 months postoperatively.

**Results:**

The reoperation rate was 3.7% for DLA, 3.0% for ALA (HRR 0.79 (0.64 to 0.99)), and 5.9% for PA (HRR 1.54 (1.37 to 1.74)). PA was associated with an increased dislocation rate compared to DLA (HRR 3.92 (3.28 to 4.67)). Fewer infections were observed with ALA (1.5%, HRR 0.68 (0.50 to 0.93)) and PA (1.6%, HRR 0.74 (0.59 to 0.92)) compared to DLA (2.2%). Similar 30-day mortality rates were found for all approaches and marginally lower one-year mortality was found for the PA. Patients operated on with the DLA reported significantly lower EQ-5D-3L index score and EQ-VAS at 12 months post-surgery compared to ALA and PA. Fewer intraoperative fractures were found using the PA.

**Conclusion:**

This study indicates that PA is associated with a higher reoperation rate after HA compared to the two other approaches. This is primarily due to high dislocation rate, despite a higher infection rate with DLA. EQ-5D-3L and EQ-VAS appear to favour ALA and PA 12 months post-surgery. Based on this study, traditional PA should be avoided in this patient group. ALA seems to be a safe alternative to the DLA.

Cite this article: *Bone Jt Open* 2025;6(10):1311–1320.

## Introduction

A hemiarthroplasty (HA) is seen as best practice when treating elderly patients with a displaced femoral neck fracture (FNF). The optimal approach when treating these patients is still debated. About 65% of HAs in patients with a displaced FNF in Norway are performed through the direct lateral approach (DLA).^1^ The DLA, as described by Hardinge et al,^2^ has been associated with an increased risk of postoperative limp, lateral pain, and of developing a postoperative haematoma compared to the anterolateral approach (ALA) and posterior approach (PA).^3-5^ Still, several studies have advocated for the DLA to be a safer choice for hip fracture patients with fewer prosthetic dislocations compared to PA.^4,6^ The PA compared to the DLA has, on the other hand, shown favourable outcomes in terms of less pain, better quality of life, and patient satisfaction.^5^ Other studies do not substantiate any difference in patient-reported outcome measures (PROMs) when comparing DLA to the PA.^7,8^ The minimally invasive (MIS) anterior approaches have been associated with faster rehabilitation and better PROMs after elective surgery with total hip arthroplasty (THA) compared to the DLA.^6,9^ However, these approaches have been criticized for reduced visibility and a longer learning curve.^10^ Ugland et al^3^ compared DLA to ALA in FNF patients and found an increased risk of abductor insufficiency in the DLA, but no statistically significant differences in PROMs.

In a scoping review, comparing DLA to PA, the authors found existing data to be highly heterogeneous and require randomized controlled trials (RCTs) or national level observational data.^11^ Therefore, by using data in the Norwegian Hip Fracture Register (NHFR),^12^ we wanted to compare three well described and frequently used approaches.

Our primary outcome was to compare the risk for reoperation within 12 months after HA for femoral neck fracture with the traditional DLA compared to ALA and PA. Secondary outcomes included one-year mortality, quality of life (EuroQol five-dimension three-level questionnaire (EQ-5D-3L)^13^ and EuroQol visual analogue scale (EQ-VAS)),^14^ and intraoperative complications.

## Methods

This is a retrospective observational study based on prospectively collected data from the NHFR.

### Patients

Data for this study were collected from the NHFR from 2005 to 2023, identifying HAs due to FNF operated through DLA, ALA, or PA.

**Fig. 1:**
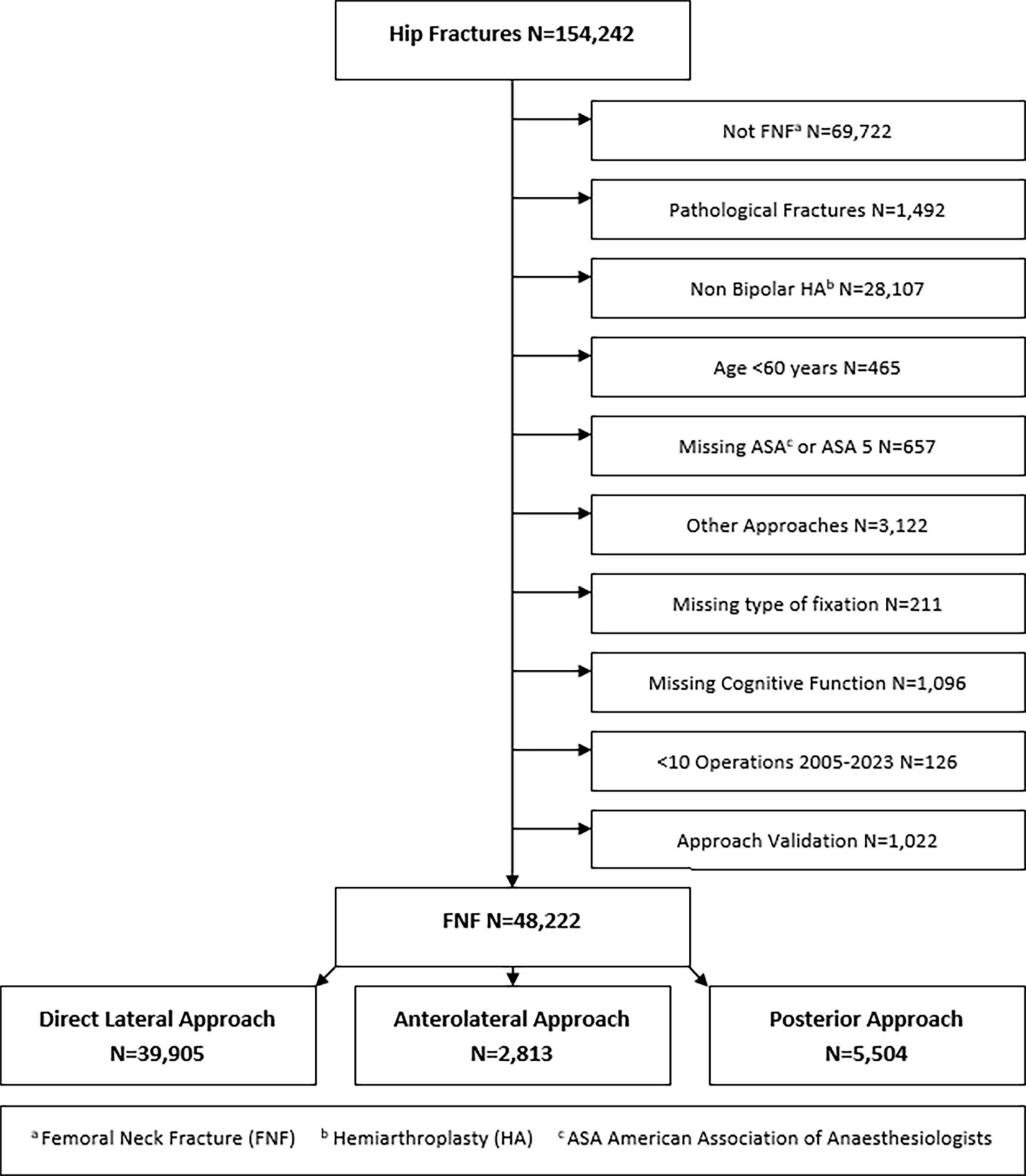
Flowchart of inclusion. ASA, American Society of Anesthesiologists; FNF, femoral neck fracture; HA, hemiarthroplasty.

A total of 154,242 hip fractures were reported to the NHFR between 2005 and 2023 (Figure 1). Only intracapsular FNFs in patients aged ≥ 60 years were included. Fractures treated with other methods than bipolar HA (screws or pins, sliding hip screw, intramedullary nails) were excluded (n = 28,107), as well as monopolar HA (n = 317). We excluded fractures in pathological bone (other than osteoporosis) and fractures with missing registration of American Society of Anesthesiologists (ASA) classification,^15^ cognitive function, surgical approach, or type of fixation of the HA (cemented vs cementless).

Preliminary analysis identified 34 hospitals using the ALA. Several of these reported only a small number of patients. To avoid misregistration, typically confusion between ALA and DLA, the senior hip surgeon or chief of surgery at each of these hospitals was contacted to clarify if the ALA had been used. After validation, 16 hospitals were found to have used the ALA within our timeframe, compared to 51 hospitals in the DLA and 39 hospitals in the PA group. Hospitals who reported fewer than or equal to ten operations were excluded. Finally, 48,222 HAs (DLA: n = 39,905, ALA: n = 2,813, PA: n = 5,504) were available for further analysis (Figure 1). The follow-up was 12 months after surgery.

ASA classification was used as a surrogate for comorbidity. Cognitive impairment was classified as present, not present, or uncertain.

### Baseline characteristics

There were small differences in age, sex, and ASA classification between patients operated with the three approaches (Table I). Cognitive impairment was more prevalent in the PA group (30% (1,652/5,504) than in the ALA and DLA groups (26% (729/2,813), and 28% (1,122/9,905), respectively. Mean duration of surgery was longer in the DLA group (77 minutes, SD 24.1) than in the ALA group (74 minutes, SD 21.9) and the PA group (70 minutes, SD 22.8). Cemented fixation was used in 83% of the prostheses in both the DLA group (33,112/39,905) and the ALA group (2,322/2,813) compared to 73% (3,993/5,504) in the PA group. There was similar distribution of surgeon experience between all three groups. Spinal anesthesia was less common in the ALA group. A larger proportion of patients was operated during regular working hours (08:00 to 16:00) in the ALA group (56% (1,570/2,813) versus 53% (21,092/39,905) in the DLA and 54% (2,967/5,504) in the PA). We found bilateral FNF in 4,753 (9.9%) of the included patients.

**Table I.** Baseline characteristics and surgical specific data.

Variable	Direct lateral approach (n = 39,905)	Anterolateral approach (n = 2,813)	Posterior approach (n = 5,504)	p-value
Mean age, yrs (SD)	82,7 (7.71)	82.5 (7.82)	83.0 (7.72)	0.025*
Female sex, n (%)	27,605 (69)	1,881 (67)	3,796 (69)	0.038†
**ASA grade, n (%)**				
I + II	12,918 (32)	904 (32)	1,836 (33)	0.317†
III + IV	26,987 (68)	1,909 (68)	3,668 (67)	
Cognitive impairment, n (%)	11,227 (28)	729 (26)	1,652 (30)	0.001†
Mean duration of surgery, mins (SD)	77 (24.1)	74 (21.9)	70 (22.8)	<0.001*
Cemented fixation of HA, n (%)	33,112 (83)	2,322 (83)	3,993 (73)	< 0.001†
Hospitals, n	51	16	39	< 0.001†
Spinal anesthesia, n (%)	35,072 (88)	2,353 (84)	4,828 (88)	< 0.001†
Daytime surgery between 08:00 to 16:00, n (%)	21,092 (53)	1,570 (56)	2,967 (54)	0.005†
Surgeon experience > 3 yrs, n (%)	26,498 (91)	2,165 (91)	3,962 (91)	< 0.001†
Intraoperative complications, n (%)	1,664 (4, 2)	104 (3.7)	135 (2.5)	< 0.001†

* Analysis of variance (ANOVA).

† Pearson’s chi-squared test.

ASA, American Society of Anesthesiologists; HA, hemiarthroplasty.

### Outcomes

The surgeons reported patient characteristics, fracture morphology, and surgical data, including intraoperative complications to the NHFR directly after surgery. Intraoperative complications were categorized as either surgical, medical, or 'other'. Surgeons reported their experience level of more or less than three years of treating hip fracture patients. Reoperations were reported and linked to the primary procedure. A reoperation was defined as any secondary procedure, including closed reduction of dislocated prostheses or soft-tissue debridement of a prosthetic joint infection. Information on patient mortality was provided by the National Population Register of Norway.

Quality of life was assessed using EQ-5D-3L and EQ-VAS at 12 months post-surgery. From June 2022, the NHFR started using the EuroQol five-dimension five-level questionnaire (EQ-5D-5L). We included only patients reporting the 3L version because of the small number of patients in the extended 5L. EQ-5D and EQ-VAS is routinely dispersed to patients directly from NHFR. The EQ-5D index score was calculated based on data from a large European dataset.^11^ Due to lack of resources, only randomly selected patients were asked to answer PROMs from January 2007 to December 2009, hence a smaller population is available for this secondary objective.

### Statistical analysis

Continuous data are presented as means (SD), categorical data as counts and percentages. We used one-way analysis of variance (ANOVA) independent-samples to compare the means of normally distributed data. A chi-squared test was used to analyze the categorical variables.

The method of Kaplan-Meier was used to estimate time to reoperation. To analyze reoperations and mortality we used a Cox regression model with adjustments for age group (60 to 74 years, 75 to 79 years, 80 to 84 years, 85 to 89 years, and > 90 years), sex, ASA grade, cognitive status, and fixation method of the prosthesis. To address potential unmeasured confounding related to clustering within hospitals, a sensitivity analysis using instrument variables was conducted. Hospital, and in a secondary model also year of surgery were used as instruments (Supplementary Material table i). Hazard rate ratios (HRRs) were reported with 95% CIs. We excluded any second hip fracture when analyzing mortality. EQ-5D index score and EQ-VAS was compared using one-way ANOVA and each dimension within EQ-5D was compared using a chi-squared test. A log-log plot was used to check for proportionality.

Statistical significance level was set at p < 0.05. The Reporting of studies conducted using observational routinely-collected health data (RECORD) statement was followed.^16^ The statistical analyses were performed using IBM SPSS Statistics v. 29.0 (IBM, USA)^17^ and the R statistical package v. 4.4.1 (R Foundation for Statistical Computing, Austria).^18^

### Ethics, data sharing plan, funding, and disclosures

The Regional Committee for Medical & Health Research Ethics South East Norway, Section C assessed the project and completed a letter of exemption (Ref 405661). The NHFR has a licence from the Norwegian Data Protection Authority (NDPA) (Ref 2004/1658-2 SVE/-). The NHFR is financed by the Western Norway Regional Health Authority. The regulations of the NDPA and the Norwegian personal protection laws prohibit the publication of the complete dataset.

## Results

### Reoperations

**Fig. 2:**
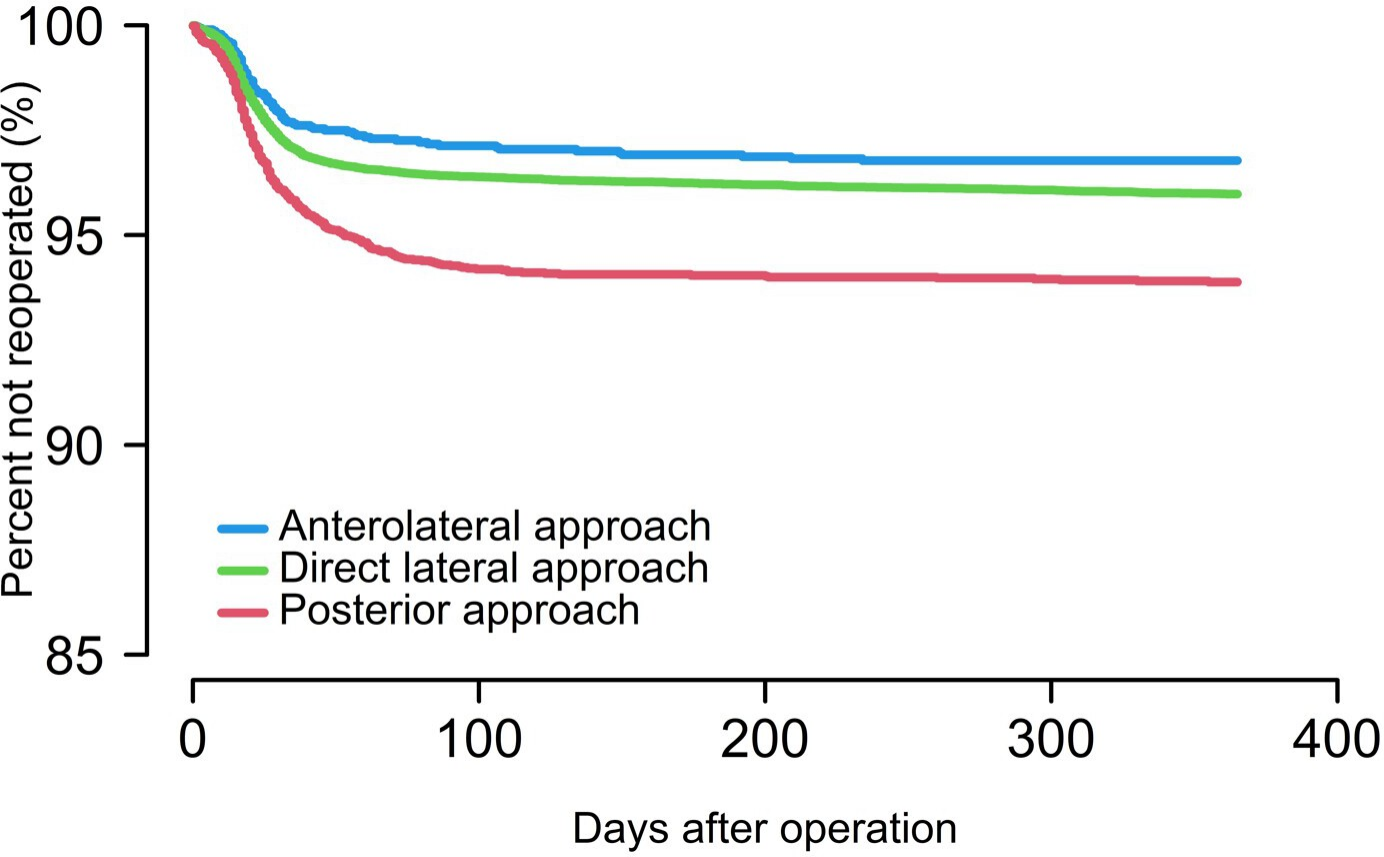
Cox regression survival curve, reoperation, and surgical approach.

The risk of reoperation was 3.7% in the DLA group (Table II). Compared to the DLA, the risk was lower in the ALA group (3.0%, HRR 0.79, 95% CI 0.64 to 0.99) and higher in the PA group (5.9%, HRR 1.54, 95% CI 1.37 to 1.74) (Figure 2). The single most important factor contributing to the difference in reoperation rates was more prosthetic dislocations in the PA. With DLA as the reference, the HRR for dislocation was 3.92 (95% CI 3.28 to 4.67) for PA and 0.87 (95% CI 0.57 to 1.34) for ALA.

**Table II.** Number and risk of reoperations after 12 months for patients operated with direct lateral compared with the anterolateral or posterior approach.

All reoperations	n (%)	HRR (95% CI)	p-value*
Direct lateral approach (n = 39,905)	1,481 (3.7)	N/A	N/A
Anterolateral approach (n = 2,813)	84 (3.0)	0.79 (0.64 to 0.99)	0.044
Posterior approach (n = 5,504)	327 (5.9)	1.54 (1.37 to 1.74)	< 0.001
**Dislocation**			
Direct lateral approach (n = 39,905)	360 (0.9)	N/A	N/A
Anterolateral approach (n = 2,813)	22 (0.8)	0.87 (0.57 to 1.34)	0.535
Posterior approach (n = 5,504)	194 (3.5)	3.92 (3.28 to 4.67)	< 0.001
**Infection**			
Direct lateral approach (n = 39,905)	862 (2.2)	N/A	N/A
Anterolateral approach (n = 2,813)	42 (1.5)	0.68 (0.50 to 0.93)	0.015
Posterior approach (n = 5,504)	90 (1.6)	0.74 (0.59 to 0.92)	0.007
**Fracture**			
Direct lateral approach (n = 39,905)	100 (0.3)	N/A	N/A
Anterolateral approach (n = 2,813)	11 (0.4)	1.54 (0.83 to 2.87)	0.174
Posterior approach (n = 5,504)	20 (0.4)	1.13 (0.69 to 1.83)	0.625
**Aseptic loosening**			
Direct lateral approach (n = 39,905)	19 (0.05)	N/A	N/A
Anterolateral approach (n = 2,813)	2 (0.1)	†	†
Posterior approach (n = 5,504)	5 (0.1)	†	†
**Other**			
Direct lateral approach (n = 39,905)	238 (0.6)	N/A	N/A
Anterolateral approach (n = 2,813)	11 (0.4)	0.74 (0.36 to 1.51)	0.404
Posterior approach (n = 5,504)	30 (0.5)	0.93 (0.59 to 1.47)	0.751

* Cox regression analyses with adjustments for age, sex, ASA, cognitive function, and fixation.

† Too few reoperations for a meaningful calculation.

N/A, not applicable.

We found lower rate of infection after ALA (HRR 0.68, 95% CI 0.50 to 0.93) and PA (HRR 0.74, 95% CI 0.59 to 0.92) compared to DLA.

Looking at all-cause reoperations, the sensitivity analysis confirmed a significantly increased risk of reoperation using the PA, compared to DLA for both models (HRR 2.3, 95% CI 2.10 to 2.56 and HRR 2.1, 95% CI 1.92 to 2.31, respectively). Comparing DLA to ALA, the instrument variable analysis showed no significant difference (HRR 0.98, 95% CI 0.78 to 1.21 and HRR 0.86, 95% CI 0.72 to 1.07, respectively).

There were similar rates of periprosthetic fracture, aseptic loosening, and other causes for reoperation between the groups.

### Secondary outcomes

**Fig. 3:**
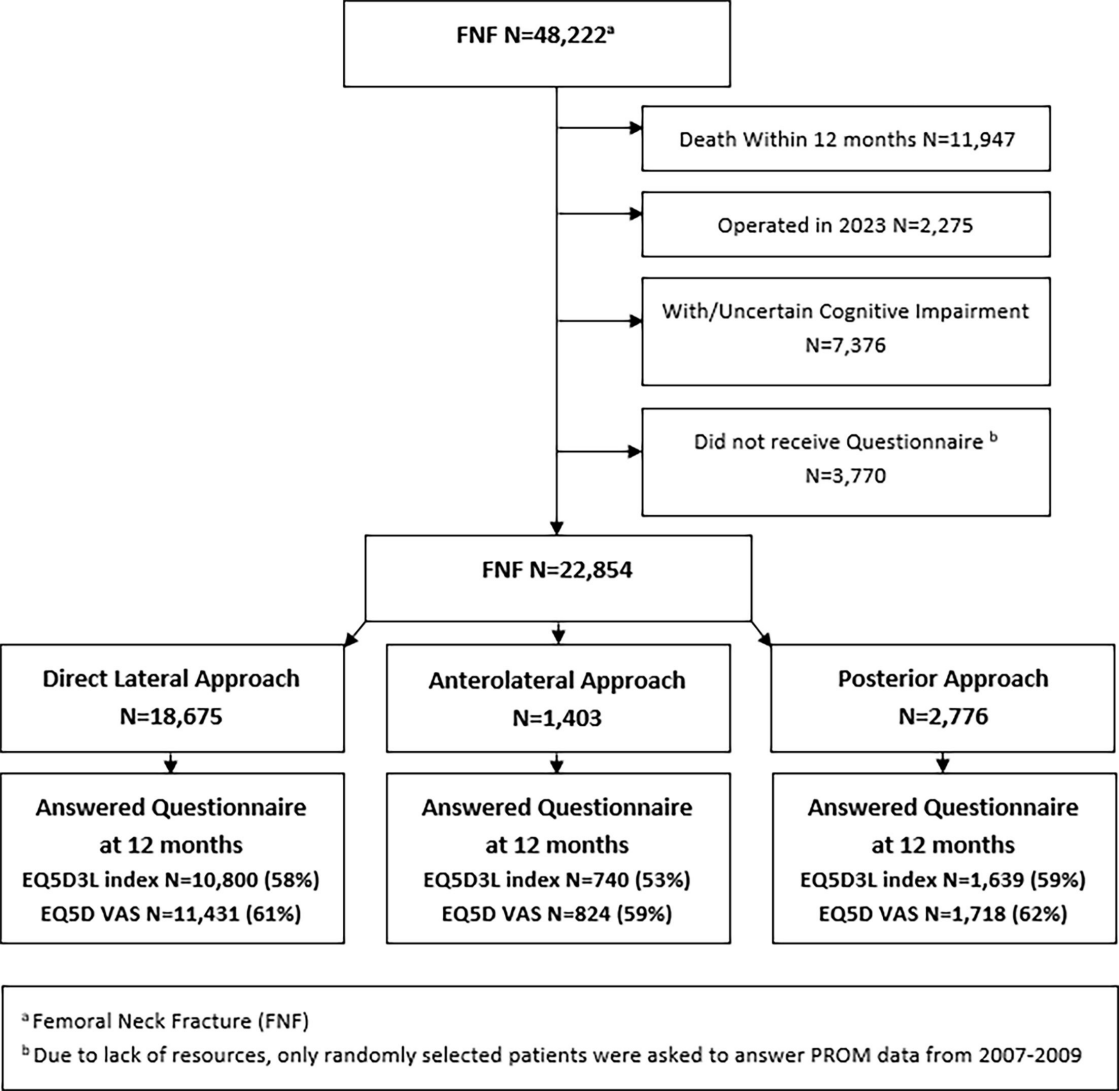
Flowchart of patient-related outcome measures. EQ-5D-3L, EuroQol five-dimension three-level questionnaire; FNF, femoral neck fracture; VAS, visual analogue scale.

There were no differences in 30-day mortality between the three groups. At one year postoperatively, there was a marginally, but statistically significant, lower mortality in the PA group compared to the DLA group (HRR 0.92, 95% CI 0.86 to 0.97) (Table III). However, this difference was not present in the sensitivity analysis for either model (HRR 0.93, 95% CI 0.86 to 1.02) and (HRR 0.94, 95% CI 0.88 to 1.01), respectively. The response rates in the three groups for the EQ-5D-3L and the EQ-VAS were 58% and 61%, 53% and 59%, and 59% and 62%, respectively (Figure 3). The EQ-5D-3L index score differed with 0.05 points favouring ALA and PA (Table IV). All five dimensions of EQ-5D-3L favoured ALA and PA over DLA. Mean EQ-VAS was 64.3 (SD 23) in the DLA group, 67.5 (SD 22) in the ALA group, and 66.5 (SD 23) in the PA group. There were fewer intraoperative fractures with the PA (n = 43, 0.8%), than with DLA (n = 499, 1.3%) and ALA (n = 39, 1.4%). Otherwise, there were only small differences in number and type of intraoperative complications between the groups (Table V). A complete overview of perioperative complications is included in Supplementary Material table ii.

**Table III.** Mortality at 30 days and one year.

Variable	n (%)	HRR (95% CI)	p-value*
**Death within 30 days**			
Direct lateral approach (n = 35,948)†	3,033 (8.4)		
Anterolateral approach (n = 2,503)†	202 (8.1)	0.97 (0.84 to 1.11)	0.641
Posterior approach (n = 4,967)†	399 (8.0)	0.91 (0.82 to 1.01)	0.089
**Death within 1 year**			
Direct lateral approach (n = 35,948)†	8,840 (24.6)		
Anterolateral approach (n = 2,503)†	609 (24.3)	1.01 (0.93 to 1.09)	0.851
Posterior approach (n = 4,967)†	1,186 (23.9)	0.92 (0.86 to 0.97)	0.005

* Cox regression analyses with adjustments for age, sex, American Society of Anesthesiologists grade, cognitive function, and fixation.

† Second hip fracture excluded.

HRR, hazard rate ratio.

**Table IV.** Patient-reported outcome measures at 12 months.

Variable	Direct lateral approach*	Anterolateral approach*	Posterior approach*	p-value
Mean EQ-5D-3L Index score at 12 mnths (SD)	0.63 (0.25)	0.68 (0.26)	0.68 (0.26)	< 0.001*,†
Mean EQ-VAS (0 to 100) at 12 mnths (SD)	64.3 (23)	67.5 (22)	66.5 (23)	< 0.001*,†
**Walking ability**	n = 11,208	n = 780	n = 1692	< 0.001‡
No problem walking about, n (%)	3,446 (31)	334 (43)	730 (43)	
Some problem, n (%)	7,501 (67)	428 (55)	926 (55)	
Confined to bed, n (%)	261 (2)	18 (2)	36 (2)	
**Self-care**	n = 11,270	n = 782	n = 1708	0.031‡
No problem with self-care, n (%)	7,090 (63)	526 (67)	1,116 (65)	
Some problem washing or dressing, n (%)	3,402 (30)	217 (28)	484 (28)	
Unable to wash or dress, n (%)	778 (7)	39 (5)	108 (6)	
**Usual activities**	n = 11,195	n = 777	n = 1693	<0.001‡
No problem performing usual activities, n (%)	3,866 (35)	325 (42)	715 (42)	
Some problem, n (%)	5,541 (50)	352 (45)	736 (44)	
Unable to perform usual activities, n (%)	1,788 (16)	100 (13)	242 (14)	
**Pain**	n = 11,244	n = 779	n = 1702	< 0.001‡
No pain or discomfort, n (%)	4,824 (43)	385 (49)	850 (50)	
Moderate pain or discomfort, n (%)	5,834 (52)	354 (45)	769 (45)	
Extreme pain or discomfort, n (%)	586 (5)	40 (5)	83 (5)	
**Anxiety/depression**	n = 11,181	n = 775	n = 1696	0.001‡
Not anxious or depressed, n (%)	7,185 (64)	522 (67)	1,166 (69)	
Moderate anxious or depressed, n (%)	3,648 (33)	230 (30)	49 (29)	
Extreme anxious or depressed, n (%)	348 (3)	23 (3)	34 (2)	

* Excluded death within 12 months, operated in 2023, cognitive impairment, no response patients.

† Analysis of variance (ANOVA).

‡ Pearson’s chi-squared test.

EQ-5D-3L, EuroQol five-dimension three-level questionnaire; EQ-VAS, EuroQol visual analogue scale.

**Table V.** Intraoperative complications.

Intraoperative complications	Direct lateral approach (n = 39,905)	Anterolateral approach (n = 2,813)	Posterior approach (n = 5,504)	Total	p-value*
**Surgically related complications, n (%**)					
Fracture/fissure of femoral shaft or greater trochanter	499 (1.3)	39 (1.4)	43 (0.8)	581	0.008
Major bleeding	84 (0.2)	8 (0.3)	14 (0.3)	106	0.609
Intraoperative death	52 (0.1)	4 (0.1)	6 (0.1)	62	0.899
Conversion from cementless to cemented fixation	15 (0.04)	1 (0.04)	3 (0.05)	19	0.834
Intraoperative conversion to hemiarthroplasty after failed ostheosynthesis	5 (0.01)	1 (0.03)	0 (0.0)	6	0.388
**Medically-related complications, n (%**)					
Cardiovascular event	192 (0.5)	9 (0.3)	12 (0.2)	213	0.013
Respiratory event	32 (0.08)	1 (0.04)	1 (0.02)	34	0.206
Anesthesia problem	241 (0.6)	12 (0.4)	14 (0.3)	267	0.003
Gastrointestinal problem	5 (0.01)	0 (0)	2 (0.04)	7	0.313
**Other, n (%**)					
Patient-related problem	32 (0.08)	4 (0.1)	5 (0.09)	41	0.545
Technical/equipment problem	249 (0.6)	12 (0.4)	21 (0.4)	282	0.046
Other	258 (0.6)	13 (0.5)	14 (0.3)	285	0.001
Total	1,664 (4.2)	104 (3.7)	135 (2.5)	1,903	< 0.001

* Pearson’s chi-squared test.

## Discussion

This study used data from the NHFR to compare three established surgical approaches in patients aged 60 years or older with a FNF treated with HA. The PA was associated with an increased risk of reoperation compared to the ALA and DLA. Dislocation was the main reason for reoperation after PA. Prosthetic joint infection was the primary cause of reoperation in the DLA, and significantly higher than for ALA and PA. In the initial Cox regression, we found a lower mortality risk one year post-surgery in the PA, but this was not reproduced in the sensitivity analyses. The EQ-5D and EQ VAS favoured ALA and PA over DLA and there were more intraoperative fractures in the DLA and ALA compared to the PA.

### Dislocation

We found a dislocation rate of 3.5% after PA compared to 0.9% in DLA and 0.8% in ALA. The dislocation rate after PA varies in the literature from 3.7% to 10.7%.^19,20^ Gill et al^20^ retrospectively reviewed 3,326 patients operated with HA for FNF and found that 3.7% of the patients operated in PA suffered a dislocation; this is similar to our study. Variations of the PA and capsule repair have not been reported to the NHFR until recently. For instance, sparing the piriformis tendon has been found to reduce dislocation rate by about 50%.^21^ The sparing pirifomis and internus, repair of externus (SPAIRE) approach may reduce dislocations rates even more.^22^ As stated by Fullam et al,^11^ 'there is a great difference in reporting of sub-variations of the PA in studies reporting on this issue, including sparing of one or more of the small rotators and posterior capsule repair'. This can explain some of the variance of reported dislocation rates using the PA. In a single centre RCT comparing DLA to PA, Parker et al^23^ preserved the piriformis and did a capsule repair including tendons of the short rotators, and found a dislocation rate of less than 2% in both DLA and PA. A Swedish registry-based study tried to avoid presumed under-reporting and linked the Swedish Arthroplasty Register to the Swedish National Patient Register.^24^ They found a 7.2% dislocation rate for the PA and 2.7% for the DLA. However, as they emphasized, this entails another source of error because the Swedish National Patient Register did not include information on laterality.

We suspect some under-reporting of closed reductions to the NHFR, but these unreported cases probably apply to all three approaches. We suspect that the under-reporting of closed reduction is a composite problem of both unawareness among surgeons that they are supposed to report closed reduction to the NHFR and the fact that in many hospitals these reoperations are performed in the emergency department outside the operating theatre. Additionally, within the Nordic Medico-Statistical Committees Classification of Surgical Procedures, there is no procedure code for closed reduction specific for HA. Furthermore, in a Danish nationwide register study on osteoarthritis patients treated with THA, one-third of the patients were miscoded for dislocation. The potential for missed dislocation probably applies to our study as well. However, Gill et al^20^ found a single closed reduction only to be successful in 16% of cases, meaning that most of the patients will need surgery for their dislocated HA, with a higher chance of entering the register due to revision surgery.

### Infection

Several papers have reported on the incidence of a postoperative infection and compared different surgical approaches. Leonardsson et al^8^ found no difference between DLA and PA in their study from the Swedish Hip Arthroplasty Register. In our study the risk of a postoperative infection was higher in the DLA. It has been hypothesized that detaching the gluteus medius and minimus from the greater trochanter increases the risk of a haematoma close to the skin.^5^ A postoperative haematoma is a risk factor for infection.^25^ Mjaaland et al^26^ compared four different surgical approaches on osteoarthritis patients operated with THA and found similar results as the present study. With DLA as reference the risk of revision due to infection was 0.57 (95% CI 0.40 to 0.8) for PA and 0.53 (95% CI 0.36 to 0.8) for ALA and anterior approach as one group.^26^ Several factors have been mentioned in the literature to increase the risk of infection; among these are higher Charlson Comorbidity score, diabetes, cancer, and vascular disease.^27^ We have no information on these comorbidities in the NHFR, but our patient groups did not differ in ASA distribution.

### Mortality

We lack a complete explanation for the marginal difference in one-year mortality. We find it unlikely to be directly influenced by the choice of approach, as the mortality rate at 30 days was not significantly different. Blom et al^28^ found an increased 90-day mortality in DLA compared to PA for primary hips, but to our knowledge this finding has not been previously reported for FNF. It is hard to determine causation for observational data and residual confounding, as differences in patient characteristics other than ASA could explain why the mortality rate is lower in the PA. The sensitivity analysis showed no significant difference between the approaches indicating that the marginally observed decreased mortality found in the PA group using the Cox model is confounded rather than showing a true causal effect.

### PROMs

In our study, there was a difference in PROMs between the groups favouring ALA and PA. The mean difference in EQ-5D-3L index score was 0.05 points. The minimal important difference in EQ-5D is not distinctly specified for hip fractures. However, Jehu et al^29^ outlined that a difference of 0.03 to 0.06 was equal to minor differences when they looked at older people with falls. The differences found in EQ-VAS between the three groups were small and probably not of clinical importance.

### Intraoperative complications

We found small differences in reported intraoperative complications, and many of these were non-specific and probably unrelated to the surgical procedure, and therefore, should not be emphasized. We did find fewer intraoperative fractures using the PA; this may be due to a difference in the mechanical forces acting on the proximal femur during surgery and femoral rasping in the DLA and ALA.

The strength of the present study lies in the large number of patients and its high external validity. We believe the results are representable also in other countries and reflect everyday clinical practice. Even though one can question the completeness of our data, the large number of patients substantiates our results.

One limitation of this study is the potential under-reporting from surgeons and hospitals, especially dislocations treated with closed reduction. The true dislocation and infection rates may be higher, but we have no reason to believe that the reporting of these reoperations is different in the three groups.

We corrected information on 1,022 patients in the NHFR that were misregistered as ALA instead of DLA. There may be a potential for misregistration in patients registered as DLA as well, but we believe this number is much smaller.

The quality of the data reported to NHFR can differ between surgeons and hospitals, including the threshold for reporting intraoperative complications. In addition, the definition of an intraoperative complication can vary between surgeons. NHFR also lacks patient-specific information which may affect their prognosis and risk of a complication; for example, smoking, alcohol abuse, and diabetes. Additionally, morphological features of the pelvis known to affect dislocation such as hip dysplasia.^30^

The difference in group size in our study can affect the risk of reporting type 1 errors because of potential imbalance in statistical power between groups and random variability may have a greater impact on the smaller groups (ALA and PA). Additionally, there will be a selection bias between PROM responders and non-responders. Previous studies have shown non-responders to be generally frailer and sicker.^31^ We categorized age into five groups. Modelling age continuously might offer higher precision, but we believe that the chosen categorization represents a reasonable trade-off between model complexity, interpretability, and epidemiological convention. Our sensitivity analysis confirmed the increased risk of revision in the PA; although the instrumental variable analysis was not performed for specific revision causes, we believe our findings may reflect true variations between the approaches. However, unmeasured confounding cannot entirely be excluded. Lastly, the NHFR report a completeness of 92% for primary HA operations and 88% for reoperations after HA.^1^ The completeness was lower in the early years of the register.

When comparing three commonly used approach for FNF, a HA performed with PA was associated with a higher dislocation rate than the other approaches. Surgeons using PA should change their practice. HAs in hip fracture patients are often performed by senior registrars on call;^1^ the DLA may be safer to perform for less experienced surgeons^4^ as it is associated with fewer dislocations. However, the DLA was associated with higher rate of infection compared to the other approaches. Our data supports the ALA as the safest choice, but this is a technically demanding surgery and the learning curve is longer. Thus, it may be difficult to change practice on a department level. Future studies should investigate if variations of the PA are as safe as the ALA and RCTs on this topic should clearly define the surgical method that is used. This will strengthen any future reviews and meta-analyses.

**Take home message**

- The risk of a reoperation within 12 months after hemiarthroplasty was 3.9% for all included patients.

- The risk for reoperation was higher when operated through the posterior approach, mainly due to periprosthetic dislocations.

- The risk of a periprosthetic joint infection was higher using the direct lateral approach.

- Patients operated with an anterolateral or a posterior approach reported better EuroQol five-dimension three-level questionnaire index score, and EuroQol visual analogue scale at 12 months, equivalent to a minor difference compared to the direct lateral approach.

## Data Availability

The datasets generated and analyzed in the current study are not publicly available due to data protection regulations. Access to data is limited to the researchers who have obtained permission for data processing. Further inquiries can be made to the corresponding author.
